# Epidemiological changes in measles infections in southern China between 2009 and 2016: a retrospective database analysis

**DOI:** 10.1186/s12879-020-4919-x

**Published:** 2020-03-06

**Authors:** Huizhen Zheng, Katherine Min Jia, Riyang Sun, Pui Hu, Maggie Haitian Wang, Benny Chung-Ying Zee, Wenjia Liang, Ka Chun Chong

**Affiliations:** 1 Center for Disease Control and Prevention of Guangdong Province, Guangzhou, China; 2 Jockey Club School of Public Health and Primary Care, The Chinese University of Hong Kong, Hong Kong, China; 3 Clinical Trials and Biostatistics Laboratory, Shenzhen Research Institute, The Chinese University of Hong Kong, Shenzhen, China

**Keywords:** Supplementary immunization, Vaccine, Child infections, Measles, China, Measles-mumps-rubella

## Abstract

**Background:**

The incidence rate of measles in China reached a nadir in 2012 after 2 supplementary immunization activities (SIAs) were undertaken in 2009 and 2010. However, the disease began re-emerging in 2013, with a high prevalence rate observed in 2013–2014 in the southern province of Guangdong. In this study, we assessed the changes that occurred in measles epidemiology during 2009–2016, particularly between 2009 and 2011 (when the influence of the SIAs were in full effect) and between 2012 and 2016 (when this influence subsided).

**Methods:**

Data from 22,362 patients with measles diagnosed between 2009 and 2016, and whose diagnoses were confirmed clinically and/or with laboratory testing, were extracted from the National Infectious Disease Monitoring Information System. Descriptive analyses were performed, and changes in epidemiological characteristics between 2009 and 2011 and 2012–2016 were compared.

**Results:**

There was a substantial surge in 0–8-month-old patients after 2012; the incidence rate increased from 4.0 per 100,000 population in 2011 (10.3% of the total) to 280 per 100,000 population in 2013 (32.8% of the total). Patients aged 0–6 years represented 73.4% of the total increase between 2011 and 2013. Compared with 2009–2011, adults aged ≥25 years accounted for a higher proportion of patients in 2013 and after (*p* < 0.01), and were highest in 2016 (31% of the patient total).

**Conclusion:**

Despite the remarkable results achieved by SIAs in terms of providing herd immunity, the 2013 resurgence of measles revealed insufficient immunization coverage among children. Therefore routine immunization programs should be strengthened, and supplementary vaccinations targeting adults should also be contemplated.

## Background

Measles is a highly contagious disease caused by the rubeola virus, which has been in continuous global circulation and has significantly contributed to the worldwide disease burden. With the widespread use of measles vaccines (MVs), the incidence rate of measles has substantially decreased in most countries. Nevertheless, the disease still causes high morbidity and mortality among children worldwide, and 95% of measles-related deaths occur in low-income countries with weak healthcare infrastructures [1].

China began deploying the MV in the 1960s and incorporated it into the routine immunization schedule under the national expanded immunization program in 1978, wherein a single dose of MV was administered free-of-charge to infants aged 8 months. Routine immunization with 2 MV doses commenced in 1985, with the first and second doses administered at the eighth month and the seventh year of age, respectively; the age of the second dose was lowered to 18–24 months in 2005 [2]. In 2006, China launched the 2006–2012 National Action Plan for Measles Elimination, following which the incidence of measles was kept at a low rate mainly owing to enhanced routine immunization coupled with supplementary immunization activities (SIAs), which were mass immunization campaigns that targeted all individuals in a specific age range regardless of their immunization history [3–5]. The purpose of the SIAs was to rapidly close the immunity gap within the targeted populations while providing herd immunity to newborns and older individuals. Unsynchronized province-wide SIAs involving MV administration were introduced in 27 provinces between 2003 and 2009 [4], and a national synchronized SIA was implemented in 2010 [3]. Although this program achieved great strides towards eradicating measles, a national resurgence of the disease occurred in 2013 along with changes in the epidemiological characteristics of measles infections [3, 6–9].

Guangdong, a province in southern China, had a high and increasing measles incidence rate before 2009, with 21.2 per 100,000 population (the highest among all provinces) in 2007 and 19.1 per 100,000 population in 2008 [10]. Despite the 2009 province-wide and 2010 nationwide SIAs, the number of measles cases began to surge in 2012 and was most notable in 2013 (accompanied by a national resurgence) [11]. Guangdong had the highest measles incidence rate nationwide in both 2012 and 2013, and the second highest in 2014, and accounted for 30, 25 and 12.8% of the nationwide patients with measles in the three years respectively [6, 7]. As the province with the largest population, Guangdong comprised 7.61% of the national population in 2011 and is critical to the country’s efforts towards eliminating measles [12]. In this study, we aimed to investigate the changes in measles epidemiology during 2009–2016, particularly in terms of how the distribution of patients by age altered between the two periods of 2009–2011 (when the effects of the SIAs were in force) and 2012–2016 (when their effects diminished). Our findings can provide the epidemiological basis for improving immunization strategies for measles elimination in the province.

## Methods

### Setting

Guangdong province has an area of 179,700 km^2^ and comprised 108 million inhabitants in 2015, 28.0 million of whom were migrants from other provinces [13]. The province hosts China’s largest floating population [14], defined as residents whose locations of household registration are in other provinces [15]. The province generally administers two doses of routine immunizations for children, with the first dose of MV (MV1) administered at the 8th month and the second dose (MV2) at 18–24 months of age [4, 16]. In 2009, all 8-month-old to 14-year-old individuals were vaccinated as part of a province-wide SIA, regardless of their immunization status. According to internal data from the Centers for Disease Control and Prevention (CDC) of Guangdong Province, approximately 20 million children were vaccinated during the 2009 SIAs, achieving an administrative coverage of 97.6%. The 2010 SIAs targeted the entire 8-month-old to 4-year-old population, with 5,603,504 children vaccinated (a coverage of 97.1%).

### Data

The measles data gathered between January 2009 and December 2016 were extracted from the National Infectious Disease Monitoring Information System (NIDMIS) by the Guangdong CDC. Two types of cases were included: clinically confirmed and laboratory test-confirmed. For laboratory-confirmed case, the blood samples collected from suspected cases within 3 days after the onset of symptoms (e.g. rash) were sent to the CDC laboratory for testing of measles-specific immunoglobulin M (IgM). The IgM test was performed using standard enzyme-linked immunosorbent assay (ELISA) method (Virion/SerionGmbH, Würzburg, Germany). Real-time polymerase chain reaction (RT-PCR) assay was used to evaluate around 10% of cases every year for a quality control. All the laboratory tests were conducted based on the internal operating procedure for measles surveillance. For clinically confirmed patients, those presenting with typical symptoms (i.e., a body temperature > 38 °C and a maculopapular rash accompanied by coryza, cough, or conjunctivitis) who were diagnosed with measles were reported to the CDC through the NIDMIS within 6 h by out- and in-patient doctors. If laboratory capacity allowed, blood samples from these patients were sent to the CDC clinical laboratories for confirmation. Serum samples were prepared according to the CDC laboratory standard operating procedures. If the laboratory capacity was insufficient, an epidemiological investigation was conducted to assess whether the suspected case had any potential exposure to other patients 7–23 days (a maximum number of 24 days in CDC practice) prior to rash onset.

The vaccination history was retrieved from the Immunization Information Management System of the Guangdong CDC, which is an electronic registry for storing individual-level immunization information for individuals born in 2005 and after. Those born in 2004 or earlier could only have their immunization records checked through paper-based hospital records or via recollection (memory).

### Statistical analysis

We conducted descriptive analyses on the epidemiological trends of measles in Guangdong during 2009–2016. The collected data included the date of disease onset, vaccination history, age, sex, household registration, and city of residence; calculations were based on incidences by age group, sex, household registration, and city of residence. Incidence was expressed as the number of reported patients divided by the group’s population size [3]. We used a heat map to represent the spatial distribution of patients in each city at the prefecture level. Changes in measles infection rates by age group, sex, and household registration were compared between the 2 periods of 2009–2011 and 2012–2016. Age was categorized into six groups: 0–8 months (although infants should have been vaccinated at the 8th months, it takes 1–2 weeks for protective antibody titers to develop [17]), 9–23 months (after having received MV1, a majority of children received MV2 at 18–24 months), 2–6 years, 7–15 years (primary and junior high school students under compulsory education), 16–25 years (senior high school students and young adults), and ≥ 26 years (adults). Vaccination histories of patients in different age groups were also compared.

We compared the proportions of cases between the 2 periods, categorized by sex, household registration and age groups, using Pearson’s chi-square tests. Statistical analyses were performed using SAS 9.4 (SAS Institute Inc., Cary, NC, USA) and the R 3.3.3 software (R Foundation for Statistical Computing, Vienna, Austria) [18]. *P*-values < 0.05 were considered statistically significant.

### Ethical considerations

This study was reviewed and approved by the Medical Ethics Committee of the Guangdong CDC. Informed consent was waived by the Committee, as the data were collected for the purpose of routine public health surveillance activities. The data were anonymized before analysis.

## Results

### Overall assessment

During 2009–2016, 22,362 cases of measles were reported to the Guangdong CDC. The annual rate of reported cases ranged between 0.3 per 100,000 population (2011) and 6.6 per 100,000 population (2013); the mean annual incidence was 2.6 per 100,000 population.

**Fig. 1:**
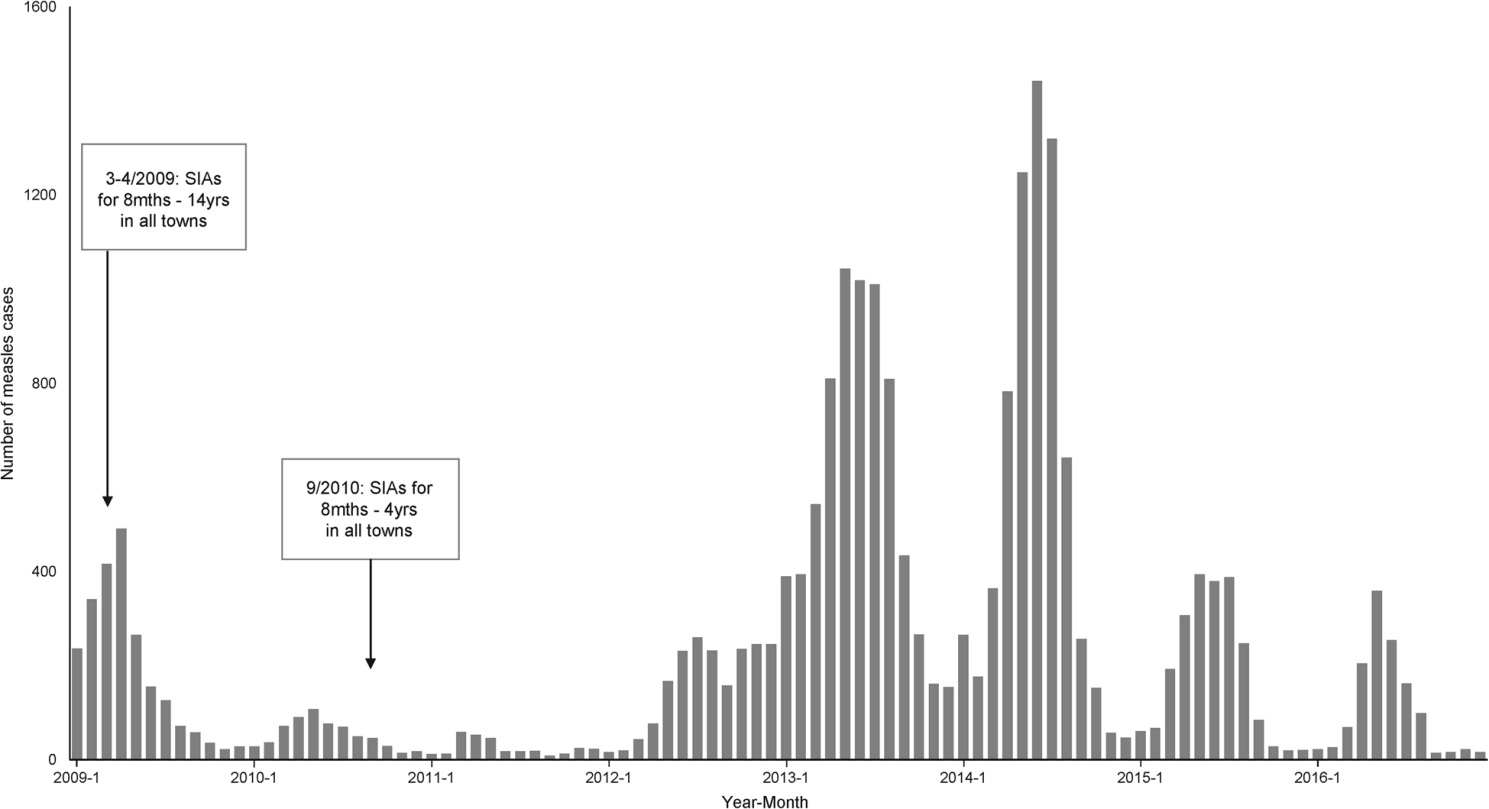
Measles monthly reported cases and province-wide immunization activities in Guangdong province (2009–2016)

Measles cases decreased by 71.5% from 2246 (2.2/100,000) in 2009 to 640 (0.6/100,000) in 2010, and reached a nadir of 308 cases (0.3/100,000) in 2011. A large-scale resurgence began in April 2012, and 1929 cases were reported that year (1.8/100,000), followed by a further increase to 7032 cases (6.6/100,000) in 2013. A monthly peak of 1442 cases was recorded in June 2014 (the highest in the study period); despite this, the annual number decreased slightly to 6752 (6.3/100,000). The number decreased again to 2190 (2.0/100,000) in 2015, where the monthly reported cases were only ranging 24–45% of their corresponding levels in 2014, and further to 1265 (1.2/100,000) in 2016 (Fig. 1).

### Distribution by city

**Fig. 2:**
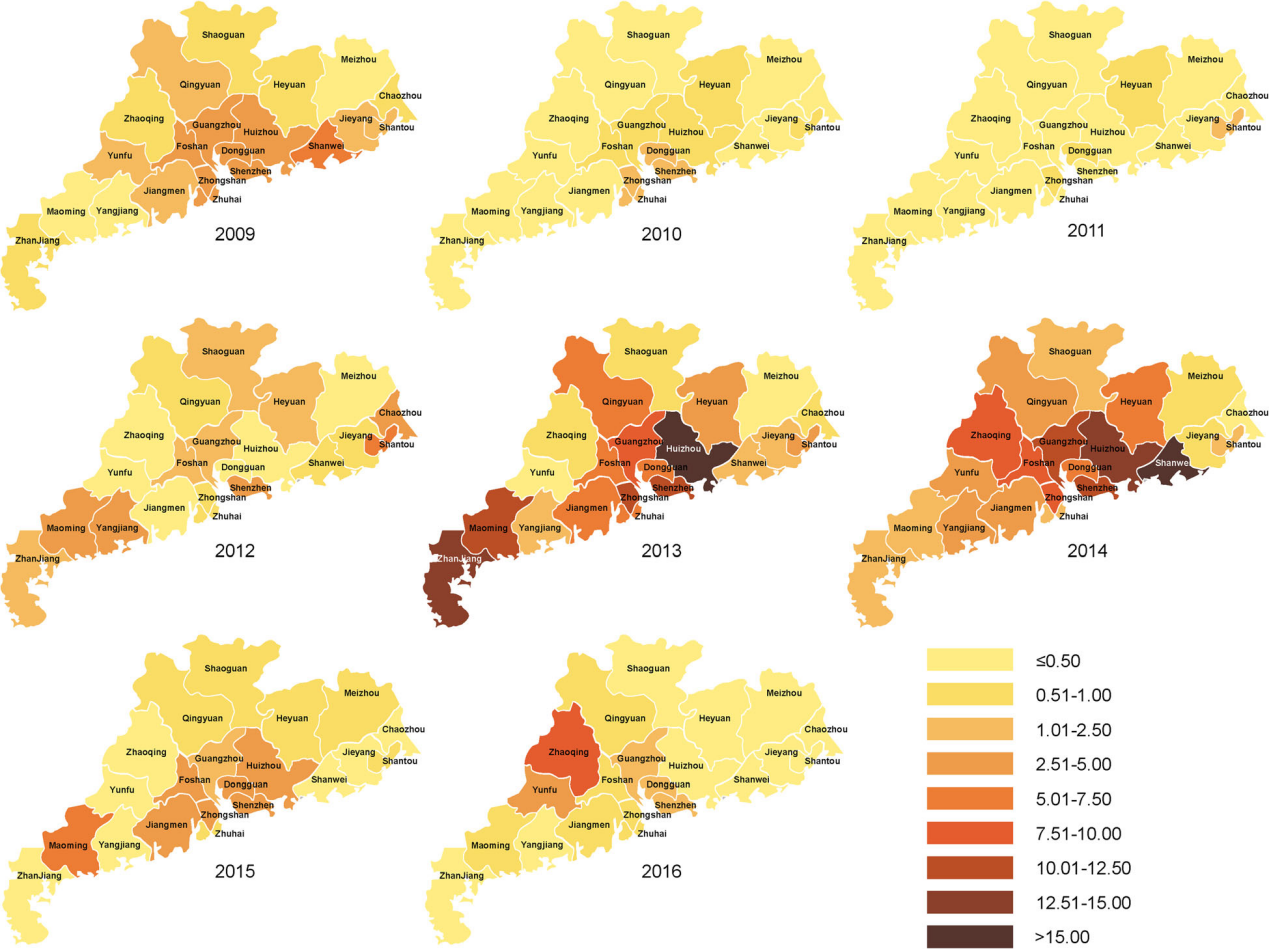
Spatial distribution of cases (per 100,000) in 21 prefecture-level cities of Guangdong

Figure 2 shows the yearly incidence in 21 prefecture-level cities in Guangdong during 2009–2016. During the resurgence, 16 of 21 cities had incidences > 1 per 100,000 population in 2013, rising to 18 cities in 2014. In 2013, 4 southern and southwestern cities contributed to 61.2% of the total increase in the number of patients since 2012: Guangzhou (*n* = 1186; 9.2/100,000), Shenzhen (*n* = 1086; 10.2/100,000), Zhanjiang (*n* = 927; 12.9/100,000), and Huizhou (*n* = 734; 15.6/100,000). Over half of the patients during 2013–2014 (54.7%) were from these 4 cities (Fig. 2).

### Distribution by sex and household registration

During 2009–2016, there were 13,117 male and 9245 female patients diagnosed with measles, with an average annual ratio of 1.4:1 (compared to 1.1:1 in the general population). The proportions of patients by sex did not change significantly between 2009 and 2011 and 2012–2016 (*p* = 0.43) (Table 1).

**Table 1:** Distribution of cases by sex, household registration and age group in 2009–11 and in 2012–16

		2009–11			2012–16		
	n (%)	Proportion of Guangdong population ^a^	Averaged incidence (Cases reported per 100,000)	n (%)	Proportion of Guangdong population ^a^	Averaged incidence (Cases reported per 100,000)	*p*-value ^b^
Overall	3194			19,168			
Sex							
Male	1853 (58.0%)	52.0%	1.18	11,264 (58.8%)	52.5%	4.01	0.43
Female	1341 (42.0%)	48.0%	0.90	7904 (41.2%)	47.5%	3.10	
Household registration ^c^							
Local	1777 (55.6%)	79.4%	0.72	13,000 (67.8%)	76.1%	3.17	< 0.01
Non-local	951 (29.8%)	20.6%	1.47	6084 (31.7%)	23.8%	4.90	
Age groups							
0–8 months	548 (17.2%)	0.8%	22.71	5862 (30.6%)	0.8%	142.24	< 0.01
9–23 months	769 (24.1%)	1.5%	16.40	4551 (23.7%)	1.7%	49.09	0.68
2–6 years	473 (14.8%)	4.8%	3.12	2646 (13.8%)	5.2%	9.61	0.13
7–15 years	315 (9.9%)	14.1%	0.63	761 (4.0%)	10.4%	1.37	< 0.01
16–24 years	558 (17.5%)	18.0%	1.03	1431 (7.5%)	19.2%	1.39	< 0.01
≥ 25 years	531 (16.6%)	60.7%	0.29	3917 (20.4%)	62.7%	1.16	< 0.01

^a^ Averaged proportion of the total population in Guangdong over the period

^b^ Pearson’s chi-square test comparing number of cases by categories between 2009 and 11 and 2012–16

^c^ Numbers of local and non-local cases do not sum up as the total number of cases because some cases had missing household registration data; chi-squared test nevertheless showed statistically significant result for cases with complete data on household registration

Regarding household registration, 30% of total cases occurred among the non-locals (i.e. migrant workers) in 2009–2016. Non-local patients generally had higher incidences than their local counterparts (Table 1). However, cases with known local household registration status increased significantly in proportion during 2012–2016 compared to 2009–2011 (*p* < 0.01).

### Age groups

**Fig. 3:**
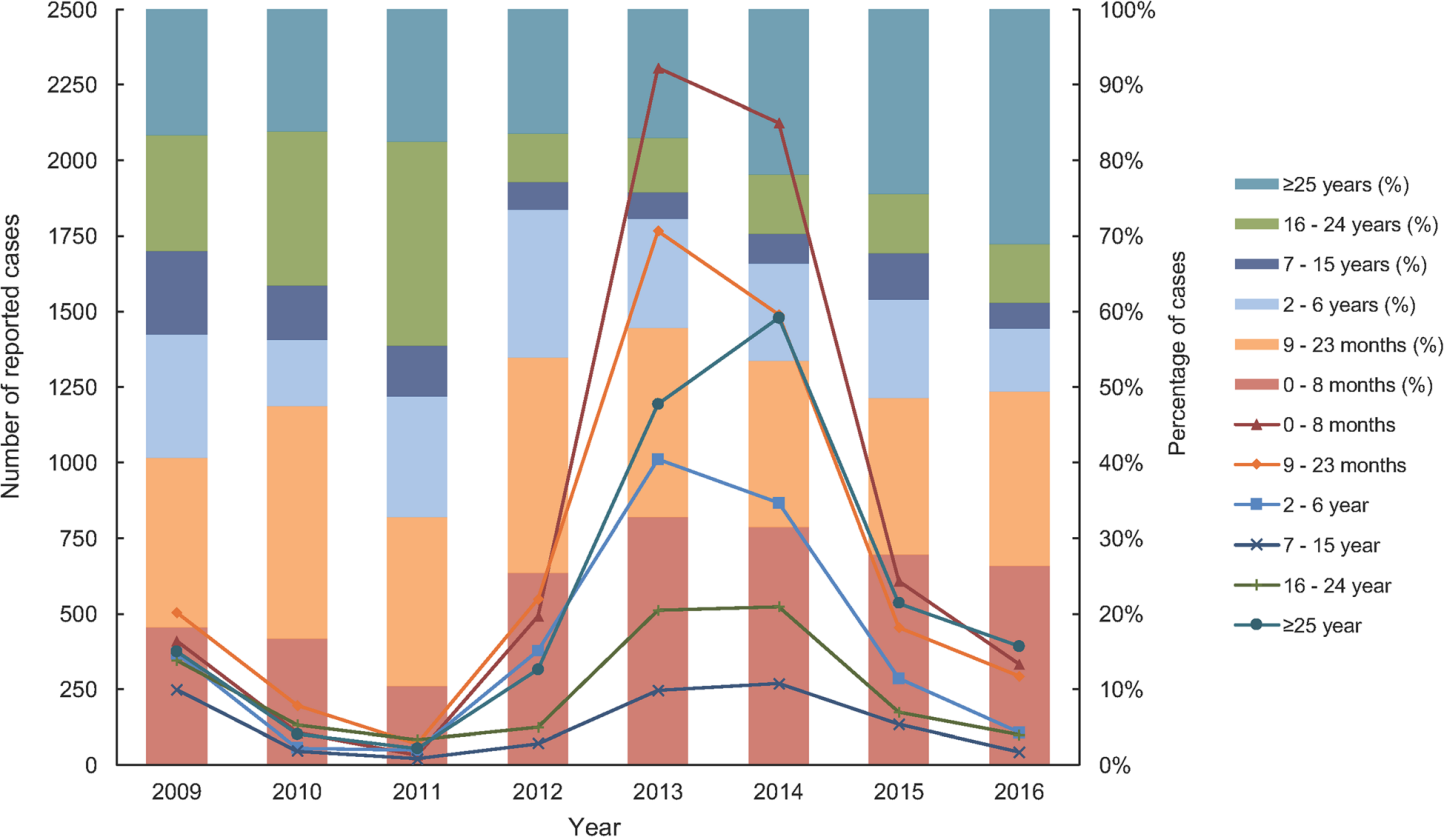
Annual number and proportion of measles cases by age groups in Guangdong

The median age at disease onset was 19 months (Table 2). The 0–8 month and 9–23 month age groups had the highest mean annual incidence, with the most number of patients, while adults aged ≥25 accounted for increasingly higher proportions of patients between 2013 and 2016 (Fig. 3).

**Table 2:** Age-specific incidences and median age of disease onset from 2009 to 2016

Age group(s)	2009 (*n* = 2246)	2010 (*n* = 640)	2011 (*n* = 308)	2012 (*n* = 1929)	2013 (*n* = 7032)	2014 (*n* = 6752)	2015 (*n* = 2190)	2016 (*n* = 1265)
Age-specific incidence(s)^a^ (cases per 100,000)								
0–8 months	49.21	14.90	4.01	62.68	279.67	254.99	76.03	37.83
9–23 months	31.06	14.23	3.90	31.30	95.60	79.73	24.51	14.29
2–6 years	7.18	1.29	0.89	7.10	18.47	15.62	5.12	1.73
7–15 years	1.39	0.34	0.17	0.63	2.25	2.44	1.21	0.35
16–24 years	1.97	0.69	0.42	0.60	2.49	2.54	0.84	0.48
≥25 years	0.64	0.16	0.08	0.48	1.78	2.20	0.78	0.57
Total	2.22	0.61	0.29	1.82	6.61	6.30	2.02	1.15
Median (IQR) age of disease onset ^b^	4 years (10 months – 20 years)	2 years (10 months – 21 years)	7 years (14 months – 22 years)	18 months (8 months – 8 years)	13 months (8 months – 10 years)	17 months (8 months – 23 years)	2 years (8 months – 24 years)	2 years (8 months – 27 years)

^a^ Age-specific incidence is expressed as the number of cases reported divided by the population size of the age group

^**b**^ Only ages < 2 years old were reported by month-age, ages ≥2 years old were reported by whole numbers

*IQR* Interquartile range

Overall, the number of patients with measles decreased rapidly across all age groups (by 75–90%) during 2009–2011 (Table 2 and Fig. 3). During 2009–2011, the 9–23 months group had the highest number of patients, and, combined with the 0–8 months group, accounted for 30–50% of patients each year (Table 1). Those aged 7–15 years, followed by those aged 2–6 years represented the lowest number of patients. The 16–24 years and ≥ 25 years groups combined represented 30–40% of the patients each year.

In 2012, measles incidence rates surged in every age group, particularly the three youngest (Table 2). During 2013–2014, the 0–8 months group comprised the highest number of patients: 2306 (280/100,000) in 2013 and 2124 (255/100,000) in 2014. The 0–8 month, 9–23 month, and 2–6 year groups comprised approximately 70% of all patients in 2012–2014, and represented 73.4% of the total increase in cases in 2012–2014 when compared to 2009–2011. The proportion of patients aged ≥25 years had also been increasing since 2013 (most remarkably in 2014), and became the highest-afflicted group in 2016 (31% of the total patients) (Fig. 3).

In the 9–23 months group, approximately 90% of patients were 9–18 months old who should have taken MV1. Thus, 82–91% of the observed yearly increases and decreases in the 9–23 months group were attributable to those 9–18 months of age. The proportion of 0–8-month-old patients increased significantly from 17.2% in 2009–2011 to 30.6% in 2012–2016, and comprised the highest number of patients overall (*p* < 0.01). A significant increase was also observed in the ≥25 years group (*p* < 0.01), while the proportions of patients decreased in the 7–15 years (*p* < 0.01) and 16–24 years (*p* < 0.01) groups (Table 1).

### Vaccination status of the patients by age group

Table 3 shows the vaccination statuses of the patients during 2009–2011 and 2012–2016. In 2009–2011, 32.6% of the patients in the 9–23 months group had received MV1. During 2012–2016, the majority of patients among the 3 youngest groups were unvaccinated, while the majority of those among the older groups had unknown histories.

**Table 3:** Proportion of different vaccination status of cases by age groups, 2009–2016

	2009–2011					2012–2016				
Age group(s)	Number of cases (n)	0 dose(%)	1 dose (%)	≥2 doses (%)	Unknown (%)	Number of cases (n)	0 dose (%)	1 dose (%)	≥2 doses (%)	Unknown (%)
0–8 months	548	66.1	7.3	0.0	26.4	5862	91.2	3.0	0.0	5.8
9–23 months	769	28.9	32.6	3.8	34.7	4551	70.7	15.1	0.4	13.7
2–6 years	473	21.1	10.1	16.5	52.2	2646	53.7	9.6	7.6	29.1
7–15 years	315	7.9	8.9	11.1	72.1	761	33.0	10.0	10.5	46.5
16–24 years	558	20.6	5.0	1.4	73	1431	17.9	4.2	1.3	76.6
≥25 years	531	16.6	2.4	0.6	80.4	3917	16.4	2.0	0.4	81.3
Total	3194	28.6	12.8	4.8	53.9	19,168	58.1	7.0	1.8	33.2

## Discussion

The incidence of measles in Guangdong Province dropped to a very low level in 1–2 years after the SIAs were introduced but resurged afterwards; this was also the trend in other regions of China [19–21]. Infants aged 0–8 months were the main group for the resurgence of measles, which was also the case nationwide [6]. As indicated by some studies, this can be explained by the 0–8-month-old patients being the most susceptible to measles outbreaks among all age groups. Although infants had immunity acquired from their mothers, most had this immunity waned in 6–8 month.

A sero-survey performed in Guangdong in 2013 revealed that only 23.58% of infants who were 4–5 months old were seropositive, as were only 10.53% of those 6–7 months old and 54.17% of those 8–9 months old [22]. Another serological study conducted in Guangdong found that infants as young as three months of age were sometimes seronegative if their mothers had low levels of measles antibodies [23]. Even women who have acquired natural immunity or been vaccinated may not transmit sufficient amounts of maternal antibodies to protect their infants during their first eight months of life. Based on these data, the majority of 6–8-month-old infants appear to be particularly susceptible to measles (i.e., have a “zero defense” status) [22, 24]. Moreover, the proportion of unprotected infants is high because mothers nowadays mostly acquire their immunity from vaccinations (especially those born after 1985 when 2-dose MVs were introduced) rather than from natural infections; hence, infants have less transmitted immunity with shorter protective durations [25, 26]. Nevertheless, a Belgian study in a nearly eliminated setting showed that maternal immunity actually waned at an early age in the children of both vaccinated and unvaccinated mothers [27, 28]; this warrants further investigation.

We conducted a further analysis of children who were too young for routine immunization (See Additional file 1). Those who were six to eight months of age comprised nearly 80% of all infants ≤8 months infected annually; a large percentage had therefore lost maternally transmitted immunity. Every group had almost the same proportion of cases during 2012–2016 as they did in 2009–2010; during the 2013–2014 resurgence, there was no increase in the proportion of infants < 6 month of age who were infected. Our evidence cannot draw conclusion on the relationship between resurgence and the trend of waning immunity, due to the lack of serological data, but the large proportion of 6–8 months old cases had indicated the need to pay attention to the immunity gap between when infants’ maternal immunity waned and vaccination administration at the eighth month. China’s immunization schedule administers MV1 at eight months of age, which is earlier than the World Health Organization’s recommendation (nine months) [29]. In general, maternal immunity that has not yet waned may interfere with the vaccine-induced immune response and result in vaccination failure [30]. Since most infants in Guangdong who are eight months have already experienced a waning in their immunity [22], 94–96% of them can successfully be seroconverted after vaccination [31]. However, advancing the MV1 delivery time to 6 months of age remains controversial [7], even though the World Health Organization suggests that infants aged ≥6 months should have a supplementary MV before routine immunization if they are in high-risk settings like daycare facilities [29]. Aside from closing the immunity gap among 6–8-month-olds, protecting unvaccinated children via herd immunity and preventing transmission from older populations should be a primary focus [7].

The second reason for the surge of measles in 0–8-month-olds was the increased number of infective individuals transmitting the virus to susceptible, unvaccinated infants. The increased infection rate among 9–23-month-olds and 2–6-year-olds were due to the lower coverage in birth cohorts after the 2010 SIAs as well as the absence of extra doses offered by the SIAs [32]. At the same time, a multi-provincial study revealed that one of the most prevalent routes of exposure was hospital visits [33]. A case-control study in Guangdong province also showed that children who visited hospitals had an odds ratio of 5.50 of developing measles within one to three weeks compared to those who did not; this was the second most significant factor after non-vaccination [34]. Moreover, infants are prone to other illness and might visit healthcare facilities more frequently for medications and regular check-ups; thus, infection control in healthcare settings is crucial for reducing the incidence of measles. In our dataset, we found that 1136 infants diagnosed with measles during 2012–2013 who were 0–8 months old (40.6%) had visited hospitals 7–21 days before disease onset, while 1478 (52.8%) had not and 183 (6.5%) had an unknown hospital visit history. The proportion of patients having been exposed to hospital settings was slightly lower than that shown in the aforementioned multi-provincial study (45%), with outpatient hospital visits being a significant predictor of infection (adjusted matched odds ratio 9.4) [33].

The rise in measles incidence rates among older age groups, especially the 9–23-month-old and 2–6-year-old groups, resulted from the aggregation of susceptible individuals after the province-wide SIAs were introduced, owing to lower vaccination coverage or primary vaccine failures. Low vaccination coverage among newly born cohorts after the 2010 SIAs was a key contributor to the national resurgence [32, 35]. A recent survey conducted in Guangdong revealed that MV1 coverage was 71.8% among migrant children aged 12–59 months, and that only 37.2% had MV1 administered in the appropriate time [36]. On the other hand, increased vaccination coverage among children ≥8 months had very positive effects on providing herd immunity to those ≤8 months. Owing to the SIAs in 2009 and 2010, incidence in every age group dropped by 60–85% in 2010; interestingly, only the 0–8- and 9–23-month-old groups had similar and substantial drops in the number of cases in 2011 as they did in 2010 (i.e., decreases of 70 and 60% respectively).

Comparing across age groups, we showed that the proportions of patients with measles aged 7–15 years and 16–24 years had dropped significantly between 2009 and 2011 and 2012–2016, as they were covered by the SIAs. Proportion of 7–15 and 16–24 years old cases were higher during 2009–2011 (9.9 and 17.5% respectively) but lower during 2012–2014 (4.0 and 7.5% respectively), indicating the effectiveness of SIAs in protecting the cohorts against outbreaks. However, similar to other Chinese provinces at the time, Guangdong experienced increasing proportions of adults with the disease from 2014 to 2016; adults accounted for the highest number of diagnoses in 2016. Relatively high proportions of adult patients were also observed after the SIAs were introduced in other studies [20, 21]. The decreasing incidence in the overall population, declining birth rate, and increasing vaccination coverage among infants and children compared to the low coverage rate in the 1980s and earlier [37, 38] all contributed to the increasing mean age of infection and build-up of susceptible individuals among adults [39]. In China, infections among adults were mainly due to susceptibility caused by missed vaccinations and decreased opportunities for acquiring immunity through natural infection [38, 40]. As revealed in our study and others, the majority of infected adults were either unvaccinated or had an unknown vaccination history [41, 42].

Various vaccination strategies have been suggested for reducing the number of susceptible individuals among infants, eligible children, and adults. First, for infants with waned maternal immunity, MV1 was not recommended at any time earlier than eight months of age on a routine immunization basis, as infants with maternal immunity that did not wane below a certain threshold would experience vaccine failure [43, 44]. An additional MV is suggested for infants six months of age in cases of outbreaks; these should be followed by MV1 and MV2 per the regular schedule [29, 45]. Second, for 8-month- and 18–24-month-old children eligible for MV1 and MV2, respectively, responsible authorities should maintain a high coverage and timely delivery of routine immunization through frequent monitoring and coverage assessment [32]. Lastly, investigations on adult susceptibility, especially in terms of secondary vaccine failure, were needed, and procedures to reduce susceptibility among adults might be warranted [42]. Although the World Health Organization and some studies have highlighted the successes of adult-targeted SIAs [46, 47], mass immunization campaigns targeting adult susceptibility were not recommended because selective and targeted SIAs; i.e., revaccination of school students, women of child-bearing ages, and populations susceptible to outbreaks, were rightfully deemed more appropriate and useful than non-selective SIAs [40, 42].

On case distribution by cities during 2013 outbreak, Guangzhou and Shenzhen had high number of cases because the two cities hosted 21.6% of total population in Guangdong and had very high proportions of migrants population [48], among whom incidence was higher and age-appropriate MCV uptake rate was low [36, 49]. As for Huizhou, a city with lower population density and fewer migrants [48], incidence among MCV-eligible children was very high; 9–11 months old cases alone accounted for 24.8% of total cases in 2013 [50]. In Zhanjiang city with low population density and a net outflow of population, incidence among the 12–23 months old was as high as 122.9 per 100,000 in 2013 [51]. The two cities also had higher percentages of cases with no or unknown immunization history (89.8% in Zhanjiang overall and 88.2% in Huizhou among 8 months – 14 years old cases) compared to Guangdong. Missing or delayed routine immunization is yet to be addressed so as to control measles in the two cities.

There were several limitations in this study. First is the possible underestimation of measles cases because some patients might not present to local doctors. Second is the limited analysis of the effectiveness of the two SIAs owing to the lack of measles data for several years before the SIAs were introduced in 2009. In our study, we focused on newly born cohorts after the SIAs (0–23-month-olds in and after 2012). Unlike those who were born in 2009–2010 and were protected by the herd immunity provided by older age groups (the 8 month- to 14-year-olds in 2009 and the 8 month- to 4 year-olds in 2010, respectively), newborns in 2012 or later had older (≥8-month-old) counterparts who did not receive additional vaccine doses administered through the SIAs. Therefore, the effectiveness of SIAs in terms of influencing the incidence of measles among newborns born within or outside their implementation periods could be assessed by examining the changes in epidemiological characteristics (e.g. age distribution) during the post-SIA resurgence in 2013. Third, the reported vaccination coverage periods for the two SIAs were only administrative estimates, and were likely slightly overestimated. Our main focus was on how newborns were protected by the herd immunity of different cohorts whether subject to the SIAs or not, and we posit that the overestimation of the administrative coverages would not appreciably affect the conclusions of our analysis since the total vaccinated population still accounted for an overwhelming majority of the population (e.g., ≥19.9 million vaccinated individuals in 2009 accounted for 93.9% of population in the eligible age range). Fourth, the routine immunization coverages during the study period were not provided to correlate with the age-specific incidence, due to possible over-estimation. Susceptibility among different age groups were discussed in correspondence with the different vaccination strategies targeting every individual in specific age groups (e.g. routine immunization for the 8 and the 18–24 months old, SIAs for the 8 months old to 14 years old in 2009), and therefore the trend of incidence in different age groups reflected effectiveness of those interventions in different age groups. Fifth, the vaccination history of the majority of individuals ≥16 years of age were unknown, although the percentage of unknown vaccination history was small among young children between 2012 and 2016; this limited our analysis on vaccination history. Lastly, the study was of limited statistical power owing to its secondary data analysis design that lacked a hypothesis-driven sample size planning.

## Conclusions

Although Guangdong province has implemented province-wide routine immunizations and SIAs with remarkably successful results, measles transmission has persisted. The 2013 resurgence of this disease revealed insufficient immunization coverage among children. Enhancing routine immunization and protecting infants from exposure are important plans of action, as infants who are ineligible for MV or eligible only for a single dose are the most susceptible to outbreaks, and contributed most to the resurgence in 2013. Future studies on the practicality of immunization strategies targeting susceptible infants and adults are required to collectively reduce infections.

## Supplementary information

**Additional file 1.** Distribution of measles cases by month-age among ≤8 month old cases in each year. A single pdf file with 1 page, the figure embedded.

## Data Availability

The original datasets used in the study area are available from the Guangdong Provincial Center for Disease Control and Prevention. Datasets were used under license for the current study, and so are not publicly available. Data are, however, available from the authors upon reasonable request and with permission of the Guangdong Provincial Center for Disease Control and Prevention.

## Abbreviations

SIAs: Supplementary immunization activities
MVs: Measles vaccine
MV1: Measles vaccine (first dose)
MV2: Measles vaccine (second dose)
NIDMIS: National Infectious Disease Monitoring Information System
CDC: Center for Disease Control and Prevention
