# Supplementary figures and images for: Epidemiological changes in measles infections in southern China between 2009 and 2016: a retrospective database analysis
Source: BMC Infect Dis. 2020 Mar 6;20:197. doi: 10.1186/s12879-020-4919-x (PMC7059666; doi:10.1186/s12879-020-4919-x)

Figure S1. Distribution of measles cases by month-age among  $\leq 8$  month old cases in each year

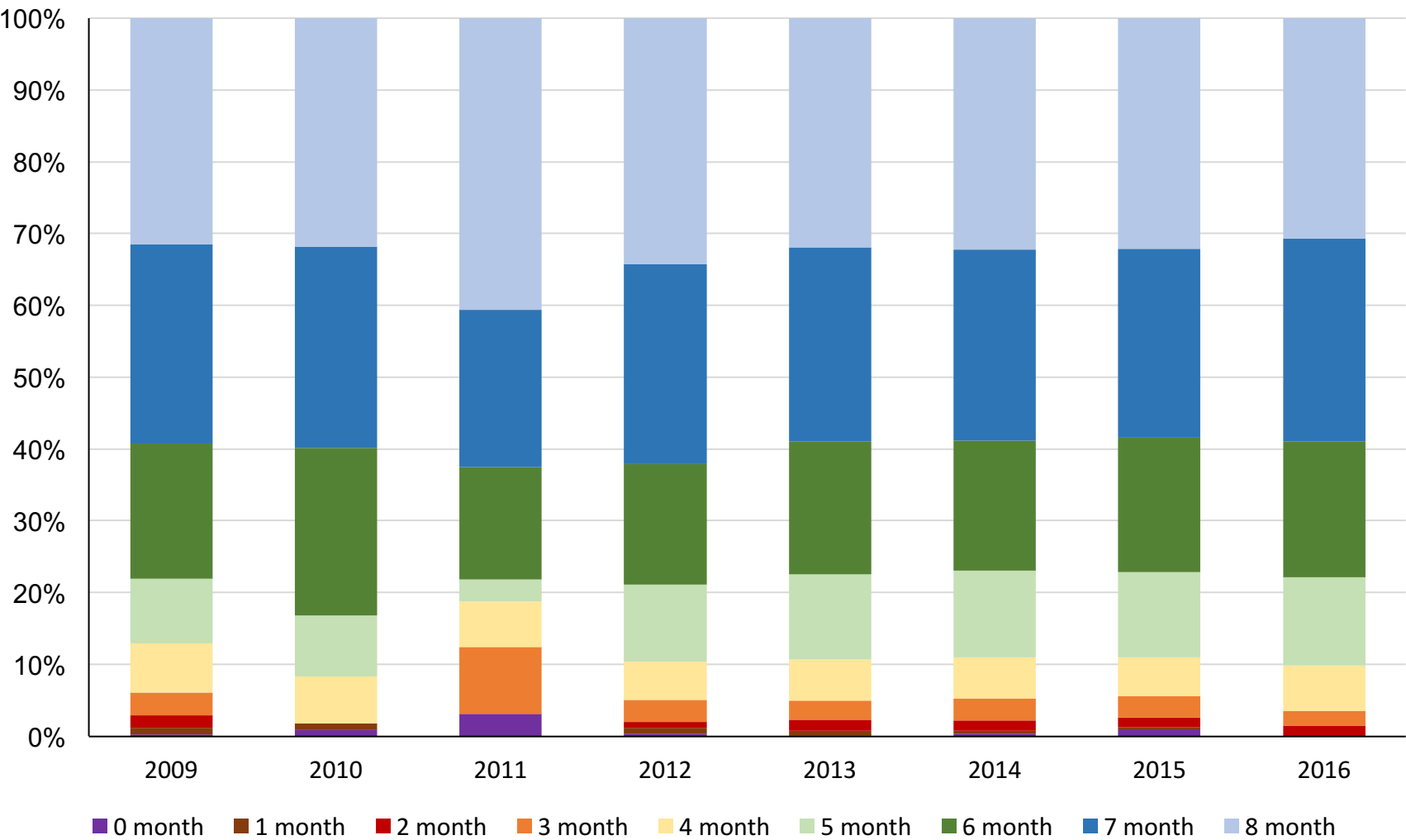

Supplement: Supplementary file 1 — Additional file 1. Distribution of measles cases by month-age among ≤8 month old cases in each year. A single pdf file with 1 page, the figure embedded. [file 12879_2020_4919_MOESM1_ESM.pdf]
